# Ultrasensitive Bead-Based Immunoassay for Real-Time Continuous Sample Flow Analysis

**DOI:** 10.3390/bios15050316

**Published:** 2025-05-15

**Authors:** Yuri M. Shlyapnikov, Elena A. Shlyapnikova

**Affiliations:** Institute of Theoretical and Experimental Biophysics of the Russian Academy of Sciences, Institutskaya 3, 142290 Pushchino, Russia; shlyapnikova@rambler.ru

**Keywords:** bead-based ultrasensitive immunoassay, magnetic beads, cholera toxin, staphylococcal enterotoxin B

## Abstract

The performance of heterophase immunoassays is often limited by the kinetics of analyte binding. This problem is partially solved by bead-based assays, which are characterized by rapid diffusion in the particle suspension. However, at low analyte concentrations, the binding rate is still low. Here, we demonstrate a further improvement of analyte binding kinetics in bead-based immunoassays by simultaneously concentrating both an analyte and magnetic beads in a compact spatial region where binding occurs. The analyte is electrophoretically concentrated in a flow cell where beads are magnetically retained and dragged along the channel by viscous force. The flow cell is integrated with a microarray-based signal detection module, where beads with bound analyte scan the microarray surface and are retained on it by single specific interactions, assuring ultra-high sensitivity of the method. Thus, a continuous flow assay system is formed. Its performance is demonstrated by simultaneous detection of model pathogen biomarkers, cholera toxin (CT) and staphylococcal enterotoxin B (SEB), with a detection limit of 0.1 fM and response time of under 10 min. The assay is capable of real-time online sample monitoring, as shown by a 12 h long continuous flow analysis of tap water for SEB and CT.

## 1. Introduction

There have been recent advances in technologies for the continuous, real-time detection of biomacromolecules, particularly vital biomarkers in vivo [1,2,3,4]. Biosensors that detect analytes in real time are expected to enable future assessments of patients’ health status and their response to treatment. However, the sensitivity of such biosensors is, in most cases, relatively low and limited to nanomolar concentrations. Only in recent years have reports emerged on achieving picomolar concentrations in real-time immunoassays [1,5]. At the same time, there are a number of important applications associated with the continuous determination of analytes in which the limit of detection (LOD) being as low as possible is desirable. This primarily concerns various biosafety applications, such as combating water or air pollution-related bioterrorism, which depends on the rapid, real-time detection of biological threat agents [6].

Recently, an ample variety of techniques based on different principles have been developed to improve immunoassay performance. These include bead-based assays, which typically alleviate diffusion limitations [7,8,9] due to the small average distance to the surface in a particle suspension. Nonetheless, many of them cannot be implemented in real time. For example, the widely used Luminex xMAP technology (Austin, TX, USA) uses pre-incubation of particles with the sample.

The use of immunomagnetic particles in a laminar flow can contribute to a sharp increase in the sensitivity of the analysis [10,11]. The efficiency of using hydrodynamic force in immunochemical and hybridization analyses on microchips using micrometer-sized beads has been known for a long time [12]. By using fluidic force discrimination (FFD) and simple counting of beads on the surface of a microchip in a microfluidic cell, it is possible to detect tumor necrosis factor at a concentration of 1 fg/mL [11]. We can summarize the undeniable advantages of these bead-linked assays: the consumption of magnetic beads in the assay is very low, they are versatile due to the possibility of conjugation with the desired specific antibody, and the beads are easily detected by an optical microscope, with each bead being able to correspond to a single intermolecular interaction [13].

Yet another attractive way to speed up the immunoassay and increase its sensitivity consists of concentrating the sample directly on the microarray, which has been reported in several publications [14,15,16]. In particular, it has been shown that electrophoretic concentration of the analyte on the microarray in a flow cell increases the sensitivity of the immunoassay by at least three orders of magnitude [17]. In this way, mass transfer limitations can be overcome, and the time required for antigen–antibody binding can be dramatically reduced. To further increase assay sensitivity, one more “active” stage can be introduced: detection of a signal in a flow of immunomagnetic particles delivering labels directly to the microarray surface in a magnetic field [15,17,18,19].

In this study, we combine the advantages of bead-based assays, which ensure continuous operation; FFD assays, for sensitive single molecule detection; and electrophoretic analyte concentration, for enhanced mass transfer. We aim to create an ultrasensitive analysis that utilizes continuous collection of the analyte on beads and its subsequent immediate detection on a microarray. To implement electrophoretic concentration on magnetic beads, we construct a special flow cell. The method is intended to be used in automatic online systems that operate without manual intervention. We demonstrate this with the detection of cholera toxin (CT) and staphylococcal enterotoxin B (SEB) in tap water.

## 2. Materials and Methods

### 2.1. Materials and Chemicals

All reagents, CT from *Vibrio cholerae* (≥90%, lyophilized powder, cat. C8052) and dialysis membrane from regenerated cellulose (cat. D9402) were purchased from Sigma-Aldrich Co. (St. Louis, MO, USA). Superparamagnetic carboxylated beads, Dynabeads MyOne, 1 µm diameter, were acquired from Invitrogen (Thermo Fisher Scientific, Waltham, MA, USA). SEB (95–98% purity by SDS-PAGE [20]) was kindly provided by Professor Yu.V. Vertiev (the Gamaleya Research Institute of Epidemiology and Microbiology, RAMS, Moscow, Russia). Monoclonal antibodies specific to CT- and SEB were described and characterized previously [17] and kindly provided by Professor E.V. Grishin and Professor F.A. Brovko [Shemyakin-Ovchinnikov Institute of Bioorganic Chemistry, RAS, Russia]. Antibodies to CT were purified by ammonium sulfate precipitation and affinity chromatography on a protein A-Sepharose column [21]. Antibodies to SEB were isolated using ammonium sulfate precipitation and chromatography on a Mono Q column [20]. Antibodies were dialyzed against mQ water and stored in 50% glycerol at −80 °C. As required, antibodies were conjugated to magnetic beads using EDC/NHS chemistry according to the manufacturer’s protocol. To ensure low conductivity during electrophoretic concentration, a buffer system of 20 mM imidazole, 10 mM glycine and 0.03% F-127, pH 9.0 (hereinafter referred to as electrode buffer), was used [17]. To prepare stock solutions of antigens, they were dialyzed against mQ water for 24 h. The concentration of the antigens was then determined gravimetrically using a quartz crystal microbalance [17]. CT and SEB solutions with a given concentration were prepared by serial 10-fold dilutions of ~1 mg/mL stock solutions with either electrode buffer or tap water from a local water supply (conductivity 700–900 µS/cm).

### 2.2. Design of Electrophoretic Flow Cell

**Figure 1:**
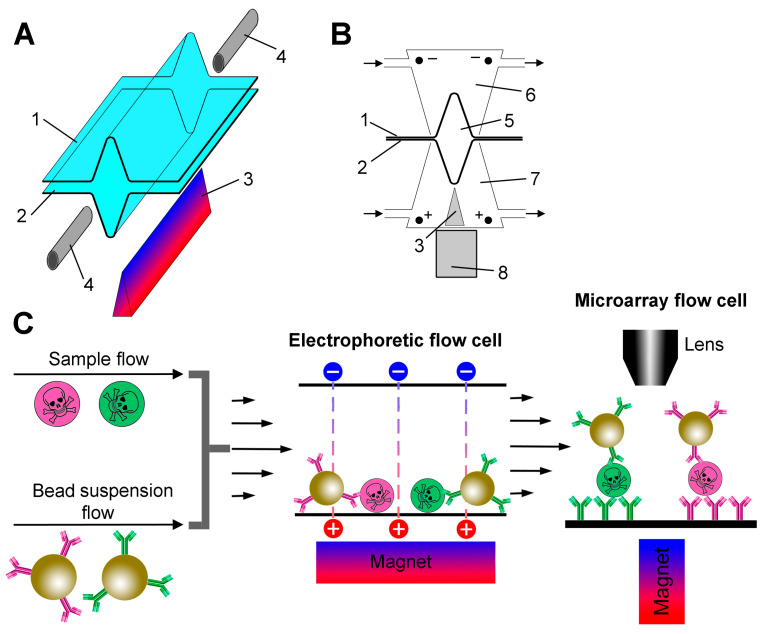
(**A**) An exploded view of the electrophoretic flow cell. Electrode chambers are not shown for clarity. The trench-shaped flow cell is formed by the upper (1) and lower (2) pieces of the dialysis membrane. A prism-shaped steel concentrator (3) is placed under the flow cell. The inlet ports (4) allow the analyte/bead suspension mixture to enter and the beads with captured analyte to exit. (**B**) A cross-section of the electrophoretic flow cell. The numbering of the parts is the same as in panel A. The flow channel diameter is ~300 µm (5). The electrode buffer flows through the upper (6) and lower (7) electrode chambers as indicated by arrows. An external magnet (8) is used to magnetize the concentrator (3) in the lower electrode chamber. (**C**) A schematic diagram of the assay process.

The design of the electrophoretic cell in two projections is shown in Figure 1A,B. Two 70 × 10 mm pieces of wet dialysis membranes were clamped together by a pair of rectangular Plexiglas blocks (70 × 30 × 10 mm) held together with screws. A channel (5) was formed between the membranes by temporarily placing a 0.3 mm diameter wire between them during the assembly of the device. The wire was removed before installing the inlets (4). The Plexiglas blocks contained chambers (6,7) with inlets for pumping the electrode buffer through them and platinum electrodes that create an electric field perpendicular to the fluid flow. The electrodes in both chambers were hermetically sealed, and the lower one was additionally equipped with a 60 × 3 × 2 mm prism-shaped steel concentrator (3) with a sharp tip to create a high magnetic field gradient. The tip was fixed at a distance of 1 mm from the flow channel. A stack of ten 5 mm sized rare earth magnets was used to magnetize the concentrator.

### 2.3. Microarray Fabrication

A dialysis membrane was treated in a radiofrequency plasma discharge in air for 15−20 s at 0.5−1 Torr. Antibody and protein G solutions (0.2 mg/mL in 3% trehalose) were printed with a spot spacing of 0.8 mm. After spotting, the microarray was kept for 0.5 h in a chamber at 100% humidity and 20 °C, then treated in a solution containing 1% sodium borohydride, 1% BSA and 0.2 M ethanolamine, pH 9.0, for 0.5 h. The microarrays were cut into 5 × 3 mm pieces with a razor and stored in 50% glycerol at −20 °C.

### 2.4. Assay Procedure

A schematic of the total experimental setup is shown in Figure S1. The total dead volume of the tubing and T-connector was about 10 µL. The microarray was installed in a flow cell whose design was described previously [18]. The length, width and height of the flow cell were 5.5, 1.2 and 0.1 mm, respectively. A small rare earth magnet ~2 mm in size was placed underneath the microarray. The sample and bead suspension (0.02% in electrode buffer) were pumped using peristaltic pumps. Thin-walled rubber tubing sections were used to dampen flow pulsations. The flow rates were 10 and 0.5 µL/min, respectively. Electrode buffer was continuously pumped through the electrode chambers at a rate of 0.6 mL/min. Immediately after the sample and bead suspension flows started, the voltage was turned on. The applied voltage was 200 V, and typical current values were 1–3 mA. The microarray surface was observed with an optical microscope equipped with a dark field illuminator and a 5 Mpx camera. Optionally, the beads were flushed from the microarray with a short (~1 s) flow pulse of 100 µL/s generated by a syringe pump (Figure S1).

### 2.5. Image Processing and Statistical Analysis

The green channel was extracted from the contrast-enhanced RGB image of the microarray. Each frame contained an image of a single spot of the microarray. The bead count per unit area was calculated in the microarray active zones and in blank areas by employing previously described custom software that counts the number of bright spots of a certain size on a dark background [22]. The LOD was defined as the minimum analyte concentration that resulted in a signal value exceeding (mean background level) + 2.5 × (standard deviation of background).

## 3. Results and Discussion

### 3.1. Design of Electrophoretic Flow Cell and Experimental Setup

The general design of the experiment is presented in Figure 1C. A schematic diagram of the entire experimental setup with its key hardware elements is shown in Figure S1. The main innovation of this work is a special electrophoretic flow cell (Figure 1A,B). In the first step, the sample flow is mixed with a suspension of beads and fed into an electrophoretic flow cell, where the beads are magnetically concentrated in a trench-shaped bottom and move along the cell by viscous force. At the same time, analyte molecules are also concentrated near the bottom surface under the action of an electric field. Thus, magnetic force, electrophoresis and viscous drag act simultaneously in a continuous process. The suspension of beads with antibody-captured analyte then enters the microarray flow cell, which is directly connected to the outlet of the electrophoretic flow cell.

The microarray flow cell is the same as described previously [18], except for the size and strength of the magnet used, as discussed below. The essence of the method is as follows. A suspension of superparamagnetic beads is pumped through a flow cell, the bottom surface of which comprises a microarray. The beads are attracted by a magnet from the suspension flow to the microarray surface and are dragged along it by the viscous force acting from the flow. The analyte-bearing beads are retained by specific antibodies immobilized on the surface, forming sandwich immunocomplexes, and are detected optically by light scattering in a dark field. By controlling the hydrodynamic force acting on the beads, it is possible to accurately discriminate between specific and non-specific adhesion of the beads, allowing individual intermolecular bonds to be detected. As shown in numerous previous publications [15,23,24,25], this technique provides exceptional assay sensitivity and specificity.

### 3.2. Assay Parameters

Several operating parameters of the electrophoretic flow cell had to be selected. Flow channel height (5) (Figure 1B) and applied voltage were close to those used in similar electrophoretic devices where the analyte is electrophoretically concentrated on a microarray [15]. These parameters are justified by the heating conditions, which impose an upper limit on the applied voltage, and by the typical values of electrophoretic mobility of the analyzed proteins. The mobility of a typical protein (albumin) at an ionic strength of 10^−3^ M and pH 8 is 2–3 × 10^−4^ cm^2^/Vs [26]. In the following calculations, we assume a uniform electric field and no aggregation of beads. We believe that these assumptions are acceptable for order-of-magnitude estimates. If a voltage of 200 V is applied to electrodes spaced ~1 cm apart, a typical analyte molecule moves at a speed of 0.1–1 mm/s and covers a channel height of 300 µm in about a second. The flow rate of ~10 µL/min is governed by restrictions imposed on the shear rate created near the microarray surface so that specific antigen–antibody bonds are not broken by the viscous drag force acting on the beads [18].

Another critical assay parameter is the bead concentration in the suspension, as it directly affects the analyte binding kinetics and, thus, the assay sensitivity. A typical bead-based xMAP immunoassay uses 6 µm beads at a concentration of 5 × 10^4^ beads/mL to bind the analyte from the sample [27,28]. The antibody conjugation density is typically 3 × 10^4^ molecules/µm^2^ [29], resulting in a total antibody concentration of 0.3 nM. Given that at low analyte concentrations the binding antibodies are in large excess, and a typical rate constant of antigen–antibody association is *k_on_* ~ 10^5^ M^−1^ s^−1^ [30], we can estimate the pseudo-first-order half-binding time of the analyte as *ln*2/(*k_on_* × [Ab]) ~ 10^4^ s. Thus, analyte binding in typical bead-based heterophase assays is slow and requires hour-long incubation.

The optimal concentration of the magnetic bead suspension is 10^−3^% or, equivalently, 5 × 10^6^ beads/mL, as discussed in Supplementary Note S1. At this suspension concentration, we have [Ab] = 10^−9^ M and a half-binding time of about 1–3 h, which is close to that of xMAP. When multiple bead types are used in the assay, binding becomes even slower, since the upper limit of 10^−3^% restricts the total bead concentration. These estimates provide a basis for accelerating analyte binding in an electrophoretic flow cell, in which local concentrations of both beads and analyte are increased by several orders of magnitude. This obviously results in significantly faster binding, allowing for shorter incubation times and lower LODs, as shown below.

### 3.3. Detection of Individual Toxins

As a model system, we used the secreted bacterial toxins SEB and CT, biomarkers of *S. aureus* and *V. cholerae*, the detection of which is of practical importance. One straightforward application is continuous monitoring of tap water for bacterial pathogens. The low conductivity of tap water allows electrophoretic concentration of analytes without overheating. The requirement for low sample conductance is the major limitation of the proposed method, as well as any other electrophoresis-based technique. Other samples, including biological ones, have a more complex composition, which may interfere with the assay. However, the matrix effect for the assays with magnetic labels was shown to be surprisingly weak: an addition of >10^10^ molar excess proteins reduced the signal by only 10–15% [15].

The microarray used for their detection consisted of three spots, two of which contained antibodies specific for SEB and CT and were located in front of the direction of bead suspension flow. The same antibodies to CT and SEB have been validated in several different types of immunoassays. The xMAP assay employing these antibody pairs has an LOD of 10 pg/mL [27], which corresponds to ~100 fM. The use of magnetic labels in combination with electrophoretic capture of toxins on a microarray allowed the LOD to be reduced to 0.1–1 pg/mL [17], while without electrophoresis, the LOD was 100 pg/mL [22].

The protein G spot served as a positive control and was located near the outlet of the microarray flow cell. The area between the active zones served as a negative control. Typically, the smallest possible spots are used in microarray design, since the smaller the sensor size, the higher the achievable sensitivity [31]. However, in our case, the analyte is collected on the beads rather than the flat sensor; therefore, the spot size does not affect the assay sensitivity. On the contrary, it is advisable to use large spots with a diameter close to the width of the flow cell so that each analyte-containing bead has the opportunity to bind to the microarray as it passes through the flow cell. After the sample mixed with bead suspension passed through the electrophoretic flow cell and the dead volume of the system, which took about two minutes, the beads with the captured analyte entered the microarray flow cell, and the signal began to accumulate (Figure S3). Then, after 5–8 min, the signal reached saturation. Thus, the response time of the described assay does not exceed 10 min, which is the sum of the dead-volume passing time (2 min) and a reasonable upper limit of the time required for the signal to reach saturation (8 min, Figure S3). We speculate that the saturation point is governed by the equilibrium between the accumulation of newly delivered beads and the shear-induced dissociation of previously bound beads from the surface. Due to the natural heterogeneity of the microarray surface, as discussed below, it contains “hot spots”—the most active binding sites, whose density is a function of the antibody immobilization efficiency. When most of these are occupied by beads, subsequent incoming beads have a lower probability of binding, and the signal ceases to accumulate. At the same time, the bead dissociation rate increases with the number of tethered beads, as suggested by the rules of first-order kinetics. A key factor governing shear-induced dissociation seems to be the mechanical strength of the antigen–antibody bond, which is related to the antibody affinity. When bead accumulation and dissociation balance each other, signal saturation is achieved.

**Figure 2:**
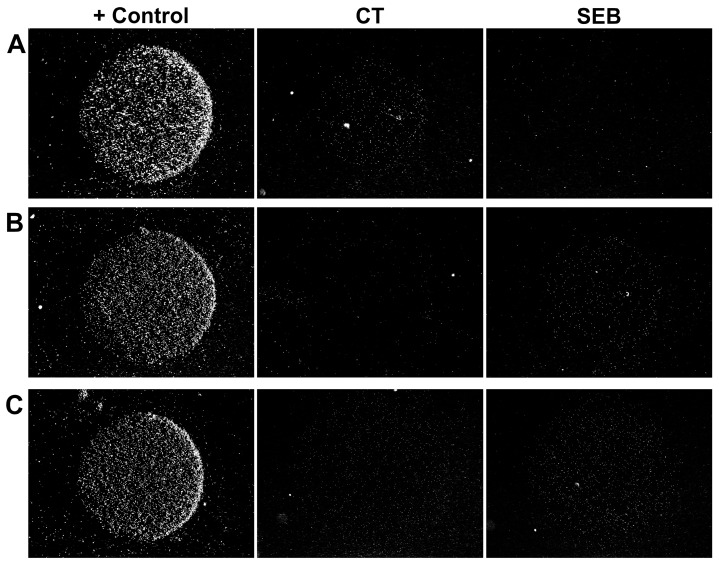
Representative examples of microarray images. The microarray comprised two specific spots with the specificity shown at the top and one positive control spot. (**A**,**B**) Individual toxins CT (**A**) and SEB (**B**), 0.1 fM each; signal accumulation time: 5 min. (**C**) Simultaneous detection of CT and SEB at 0.1 fM each; signal accumulation time: 8 min. Each image shows a 1.3 × 1 mm^2^ area.

**Figure 3:**
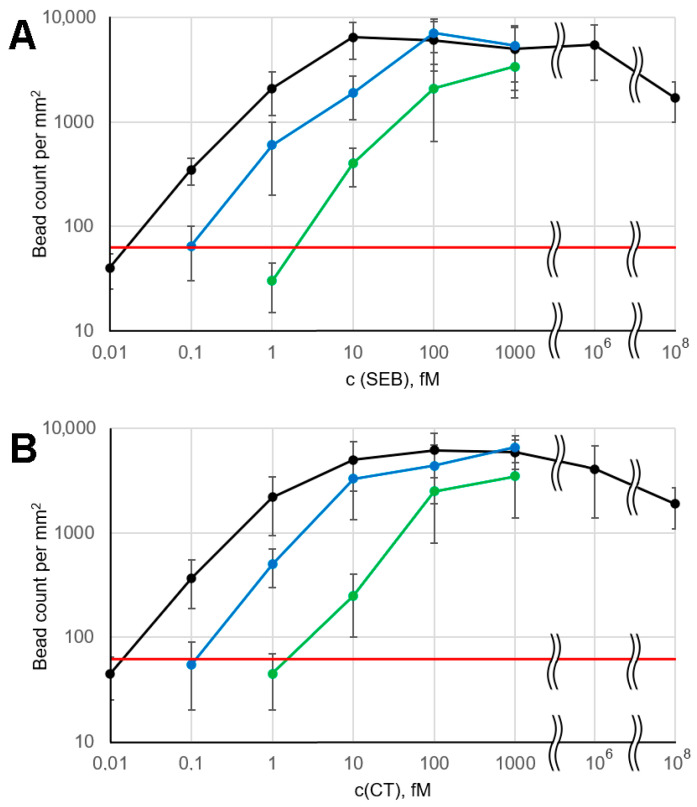
Calibration curves under various assay conditions: black—with applied electric and magnetic fields, green—with switched-off electric and magnetic fields in the electrophoretic flow cell, blue—with electrophoretic flow cell replaced by 300 × 2 mm tubing. (**A**) SEB; (**B**) CT. The red line marks the mean background + 2.5 STD. Error bars correspond to 2.5 × STD.

For both analytes, the obtained LOD value was 0.1 fM or ~10^5^ molecules/mL, as shown by the experimental data in Figure 2 and their quantitation in Figure 3. At an analyte concentration of 0.01 fM, the signal was statistically indistinguishable from the background, indicating the absence of non-specific binding. At first glance, such extremely low LOD values seem implausible due to the following quantitative estimates. It was previously assumed that in a strong magnetic field, beads do not rotate, so that only ~1% of their surface contacts the microarray [18]. When analyzing an analyte solution at 0.1 fM, 5000 molecules pass through the microarray flow cell in 5 min of signal accumulation, of which only 1% can be detected, since the remaining 99% are captured in inaccessible areas of the bead surface. That is, no more than 50 beads should be detected in the corresponding active zone, but the experimentally observed signal value often exceeded this number (Figure 2 and Figure 3). We speculate that this discrepancy can be attributed to the rotation of the beads in a weak magnetic field. As mentioned above, we used a small magnet in the microarray flow cell instead of the previously used large one [18]. Accurate modeling of the magnetic field in the flow cell was beyond the scope of this work, so we performed an empirical optimization. The key conditions that the magnetic module had to meet were as follows: (1) no interference with the dark field illumination of the beads and (2) a sufficient magnetic field gradient across the flow cell to ensure that the beads are efficiently attracted to every part of the microarray surface. The previously described geometry [18] (Figure S4A), for which no bead rotation was shown, satisfied both of these conditions, but the magnetic field was highly excessive. It was found that the minimum acceptable magnet size was ~2 mm, which is ~3 times smaller than the flow cell length, and its minimum distance to the surface was about 1.5 mm (Figure S4B). Further decreasing the magnet size (or increasing its distance to the microarray) resulted in poor attraction of the beads near the flow cell inlets. Reducing magnetic field strongly reduces the torque resisting bead rotation, which is proportional to the square of the magnetic field induction [32]. Thus, we suggest that our experimental setup provides some freedom of bead rotation, increasing the portion of the bead surface accessible for contact with the microarray. This hypothesis was supported by experiments comparing the assay performance with large and small magnets for both analytes studied (Figure S5). With the large magnet, the LOD increased tenfold to 1 fM, and the entire calibration curve shifted by an order of magnitude toward higher concentrations. This fact indicates a substantial decrease in bead surface accessibility in a stronger magnetic field, which locks the rotational degrees of freedom of the beads.

### 3.4. Multiplex Assay

The results of the multiplex assay of a sample containing 0.1 fM SEB and 0.1 fM CT are presented in Figure 2C. It can be seen that the signal values are comparable to those for the single analyte assay (Figure 2A,B), given that the signal accumulation time is the same for each bead sort. Thus, multiple sorts of beads can be used simultaneously if the antibodies are sufficiently specific. In this work, we used antibody pairs that had already been validated and showed no cross-reactivity [17]. Nevertheless, in this study, we tested these antibodies for cross-reactivity in the new assay format. No non-specific signal was detected, even at high analyte concentrations (Figure S6). We thus demonstrated that the method is suitable for multiplexing on par with other microarray-based assays. The upper limit on the number of simultaneously detected analytes in the proposed assay is determined by the total bead concentration in the suspension and the number of active zones in the microarray. We do not see any fundamental limitations for the simultaneous analysis of up to ten analytes.

### 3.5. Calibration Curves and Electrophoresis Effect

Large active zones allow for ample bead count statistics and simplify image processing. The calibration curves (Figure 3, black lines) exhibit the general features typical of microarray-based immunoassays with magnetic labels, including a relatively narrow dynamic range [17,24]. No hook effect was observed at analyte concentrations up to 1 nM, likely due to the relatively high binding capacity of the magnetic beads: the effective concentration of antibodies conjugated to the beads, as estimated above, was approximately 1 nM. The hook effect was detected only at high antigen concentrations (100 nM SEB and CT), obviously due to substantial blocking of the microarray binding sites by the free analyte. However, such a high concentration of bacterial toxins (corresponding to ~10 µg/mL) seems unrealistic in practical applications. We attribute the weak hook effect to the fact that the magnetophoretic transport of bead-bound analyte is much more efficient than the corresponding competing process, namely diffusion of free analyte to the microarray surface.

To demonstrate the benefits of employing an electrophoretic flow cell, we performed several control experiments: (1) with both the electric and magnetic fields turned off, and (2) with the entire electrophoretic flow cell replaced by 2 mm diameter tubing, which increased the incubation time of beads with the sample from 2 min to approximately 1.5 h. In both cases, a significant decrease in assay performance was observed (Figure 3). According to the above estimates, even a 1.5 h incubation time does not reach the typical half-binding time, resulting in lower signal values at a given analyte concentration. When the electrophoretic flow cell is used with electric and magnetic fields turned off, the total incubation time is only about 2 min, which is determined by the system’s total dead volume. This time corresponds to a ~1% binding reaction depth. Thus, the calibration curves shift by two decimal orders toward higher concentrations compared to the electrophoresis-assisted assay (Figure 3).

The data presented in Figure 2 and Figure 3 provide experimental evidence for the ultra-high sensitivity of the assay. A statistically significant positive signal was observed in each of the triplicate repeats of the 0.1 fM SEB and CT assay. Thus, the 0.1 fM value represents an experimental upper limit of the LOD, while the extrapolated value may be even lower. Its precise determination is complicated by the large signal variations that are common in assays with magnetic labels, as discussed below. Also, the observed LOD is much lower than that of most commonly used assays, such as ELISA, including its ultrasensitive amplified version [33]. This makes the validation of our technique against “gold standard” methods challenging and beyond the scope of this work.

### 3.6. Real-Time Online Sample Monitoring for Biomarkers of Bacterial Pathogens

The LOD and response time achieved with the method presented here are comparable to those of several previously described assays [24,25]. In this section, we demonstrate the fundamentally new advantages that the proposed technology provides. Unlike other heterophase assays, in this case, the microarray is not used to capture the analyte from the sample but serves solely as a signal detector than discriminates between beads with and without bound analyte. This makes our method similar to flow-based assays such as xMAP, in which the analyte bound to specific beads is revealed by continuously passing a bead suspension through a signal detector. However, FFD-based detection on microarrays is more sensitive than traditional fluorescence detection: even without electrophoresis (Figure 3), our technique achieves an LOD at least 100-fold lower than that of xMAP using the same antibody pair [27].

After a signal is acquired by imaging of the bead-decorated surface, the beads can be flushed from the surface with a short (~1 s) 100 μL/s flow pulse generated by a syringe pump (Figure S1). This pulse generates a shear rate high enough to cause each bead to break away from the microarray surface, leaving it intact. Thus, there is no need to replace the microarray after each analysis, and the entire system can operate for a long time with a single microarray installed in the flow cell. This makes the assay well suited for continuous, real-time monitoring of samples. To demonstrate this capability, we performed multiple 12 h experiments, each including 144 cycles of 5 min signal accumulation, microarray imaging and bead flushing.

**Figure 4:**
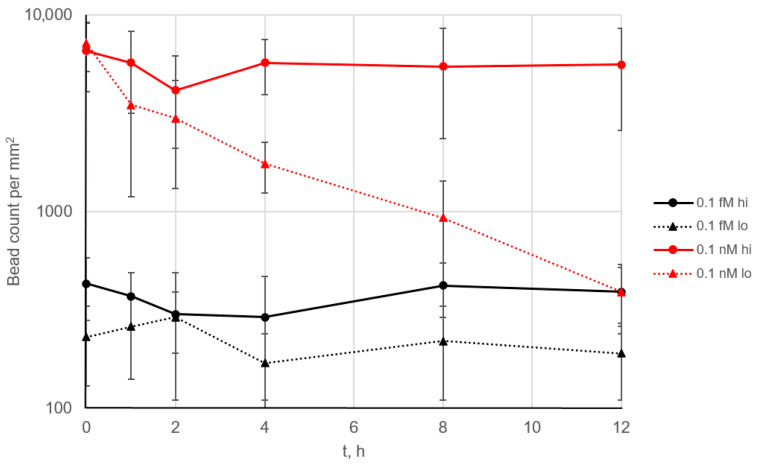
The long-term performance of the continuous assay. The SEB-specific signal over time is shown for 0.1 fM (black) and 0.1 nM (red) SEB. Each 5 min cycle included signal accumulation, imaging and flushing (Figure S1). The background level was below 100 beads/mm^2^ (Figure 3). “Hi” and “lo” indicate high- and low-affinity antibodies conjugated to the beads, respectively. Error bars correspond to 2.5 × STD.

When 0.1 fM and 0.1 nM SEB solutions were used as a model system, no signal decrease was observed across the 144 cycles (Figure 4, solid lines). This demonstrates complete regeneration of the surface after each cycle and indicates the absence of any noticeable degradation of the microarray, such as a loss of antibody activity or its detachment from the surface. In addition, even at the relatively high analyte concentration of 0.1 nM (10 ng/mL), no free analyte remained in the solution, which would otherwise block the binding centers on the microarray.

The affinity constants of the antibodies were measured previously [17]. The bead-conjugated antibody had higher affinity (association constant 2.5 × 10^9^), whereas the microarray-bound antibody had significantly lower affinity of 4.5 × 10^8^. When the bead was torn off from the surface, the weakest bond in the antigen–antibody sandwich complex was broken; in our case, this was the bond between analyte and the microarray. However, the situation changed when the antibody pair was reversed, i.e., the higher-affinity antibody was immobilized on the microarray (Figure 4, dotted lines). While no signal decrease was observed at low analyte concentrations, at 0.1 nM, the signal dropped more than 10-fold over 12 h. In this case, the analyte likely remained bound to the microarray after the beads were removed. At low analyte concentrations, this effect may not result in a significant reduction in the density of analyte binding sites, but at higher concentrations, it becomes more pronounced.

Thus, a fundamentally novel effect can be assumed: analyte molecules that have previously bound to magnetic beads can block the binding sites on the microarray and remain on its surface after washing. This finding suggests that additional care should be taken when optimizing antibody pairs for this type of assay: the binding constant of the analyte to the antibody on the beads should be higher than that to the antibody on the microarray.

## 4. Conclusions

In this paper, we describe a new type of ultrasensitive heterophase immunoassay. Its main innovation is a multifunctional flow cell in which electric, magnetic and viscous forces act simultaneously, allowing for fast and efficient binding of the analyte to magnetic beads in continuous flow mode. This feature differentiates our method from previously published ones. In this sense, it is the pinnacle of the “active assay” principle, which employs directed force to enhance mass transfer, resulting in increased speed and sensitivity of analysis [14,15,18]. Fluidic force discrimination [10] on the microarray surface is then used to rapidly detect the bead-bound analyte. To confirm the effectiveness of the developed method, a multiplex analysis of SEB and CT was carried out. The achieved LOD was 0.1 fM, and the response time was about 5 min. Although various methods and strategies for the detection of bacterial toxins, including SEB, have been reported [17,34,35], the main advantage of the proposed technique compared to previously described assays is its suitability for continuous online sample monitoring. It employs inexpensive hardware and consumables. The only costly reagent is a suspension of antibody-coated magnetic beads, but its consumption is extremely low: 0.6 µL of 1% suspension per hour. Combined with its high sensitivity and short response time, this makes the assay ideally suited for various biosafety applications. In particular, it can be used in fully automated devices, since it allows continuous real-time sample processing without any manual intervention, such as replacing the microarray. In this paper, the proposed concept was illustrated by monitoring tap water for common bacterial pathogens, but many other applications are also feasible.

Finally, we should discuss the key issue of the proposed technique, namely its poor accuracy of analyte quantitation due to high signal variations and low dynamic range. A lack of reproducibility and high variability have been an issue in conventional microarray-based assays for many years, one of the main sources of which being surface inhomogeneity [36]. For heterophase assays with magnetic labels, this problem is even more pronounced [17,22,25], since not a single microarray–solution interface but a pair of contacting surfaces (bead and microarray) is involved in the assay process. Additional sources of variability are flow and magnetic field inhomogeneities in the flow cell. These factors restrict the precision of quantification to one decimal order.

Several approaches have been proposed to overcome the problem of variability [24]. They include (1) internal standardization by relating the signal intensity in a specific spot to that of a nearby reference spot and (2) signal measurement in a narrow linear range of the calibration curve, where its high slope diminishes the effect of signal variation. The applicability of these approaches to the assay described here is the subject of further research.

## Data Availability

The original contributions presented in this study are included in the article/Supplementary Materials. Further inquiries can be directed to the corresponding author.

## Supplementary Materials

The following supporting information can be downloaded at https://www.mdpi.com/article/10.3390/bios15050316/s1, Figure S1: A schematic and photo of the experimental setup. Supplementary Note S1: Selection of the optimal bead suspension concentration. Figure S2: Signals in the model immunoassay as a function of the magnetic bead suspension concentration. Figure S3: SEB signals and background accumulation kinetics for the indicated analyte concentrations. Figure S4: Designs of magnet placement in the microarray flow cell. Figure S5: Calibration curves obtained with large and small magnets. Figure S6: An illustration of the assay specificity.

## Abbreviations

The following abbreviations are used in this manuscript:

CT	Cholera toxin
SEB	Staphylococcal enterotoxin B
LOD	Limit of detection
FFD	Fluidic force discrimination
