# Supplementary material for: Ultrasensitive Bead-Based Immunoassay for Real-Time Continuous Sample Flow Analysis
Source: Biosensors (Basel). 2025 May 15;15(5):316. doi: 10.3390/bios15050316 (PMC12109733; doi:10.3390/bios15050316)
Supplement: Supplementary file 1 [file biosensors-15-00316-s001.zip › biosensors-3575096-supplementary.pdf]

Supplementary Materials

Ultrasensitive Bead-Based Immunoassay for Real-Time Continuous Sample Flow Analysis

Yuri M. Shlyapnikov * and Elena A. Shlyapnikova

Institute of Theoretical and Experimental Biophysics of the Russian Academy of Sciences, Institutskaya 3, 142290 Pushchino, Russia; shlyapnikova@rambler.ru

* Correspondence: yuri.shlyapnikov@gmail.com

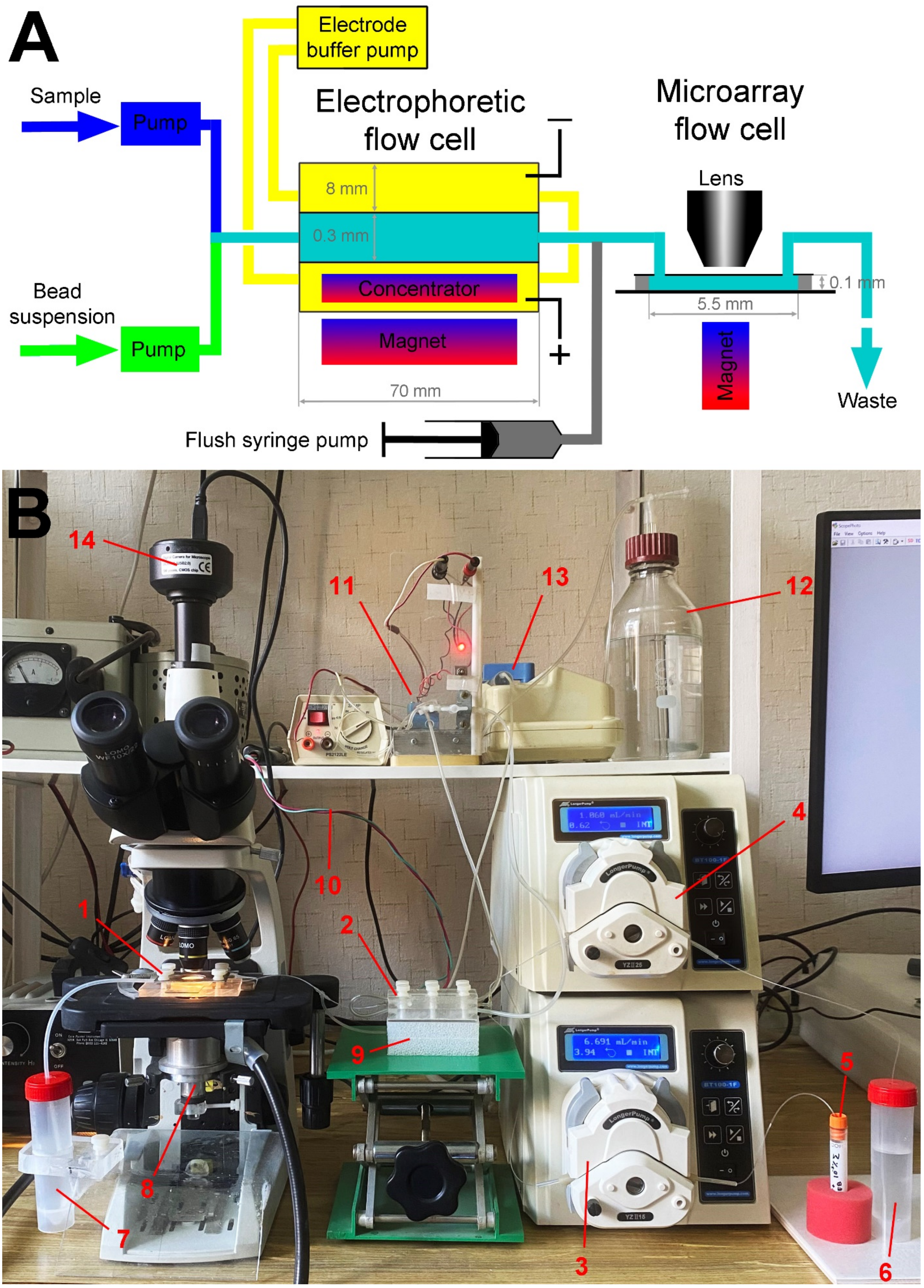

Figure S1. A schematic (A) and photo (B) of the experimental setup: 1—microarray flow cell; 2—electrophoretic flow cell; 3—bead suspension pump; 4—sample pump; 5—bead suspension vial; 6—sample vial; 7—waste vial; 8—illumination and magnetic module of the microarray flow cell; 9—electrical insulation and magnet casing of the electrophoretic flow cell; 10—conductors to 200V direct current power supply; 11—electrode buffer pump; 12—electrode buffer flask; 13—flush syringe pump; 14—camera.

Supplementary Note S1. Selection of the optimal bead suspension concentration.

It was previously found that the empirical value of the optimal concentration of 1 μm magnetic bead suspension in a microarray-based assay with magnetic labels is 10^{-3} % [17]. Here, we discuss the rationale for this value. Decreasing the bead concentration obviously results in an increase in the signal accumulation time. Moreover, at some low bead concentrations, the dissociation of beads from the microarray can counterbalance the influx of newly delivered beads. Thus, the highest possible bead concentration is preferred. However, concentrations above a certain threshold lead to the aggregation of magnetized beads into chains. This process is autocatalytic since bead aggregates are stronger magnets than individual beads. Also, it significantly reduces the assay performance, since the viscous force acting on a chain of uncontrollable length is much higher than that acting on a single bead, and a single antigen–antibody bond is no longer able to retain the bead on the surface. Although quantitative modeling of this process was beyond the scope of this work, we performed direct experiments to empirically demonstrate the optimal bead concentration. We studied a model assay system in which CT at a concentration of 10^{-3} mg/mL was directly spotted onto the microarray as described in the Materials and Methods, and the microarray was scanned with different concentrations of CT-specific beads. As shown in Fig. S2, a catastrophic decrease in the signal was observed below 10^{-3} %. Thus, we considered 10^{-3} % as an empirical upper limit at which bead aggregation was about to begin and used this concentration in further experiments.

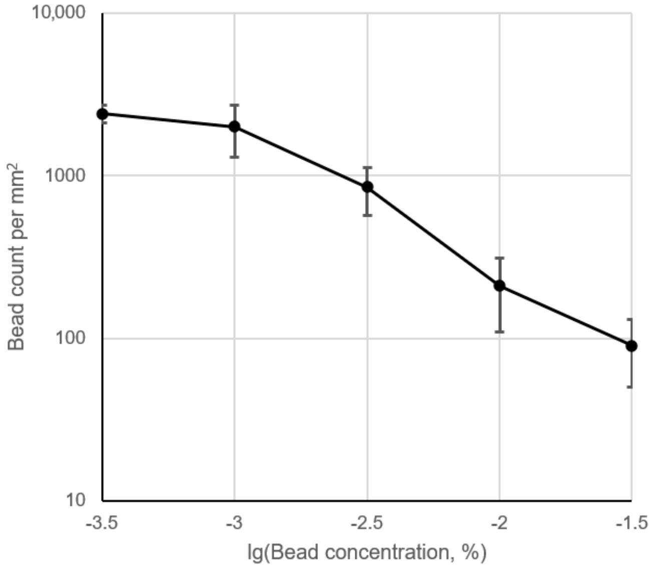

Figure S2. Signals in the model immunoassay as a function of the magnetic bead suspension concentration. Error bars correspond to 2.5 × STD.

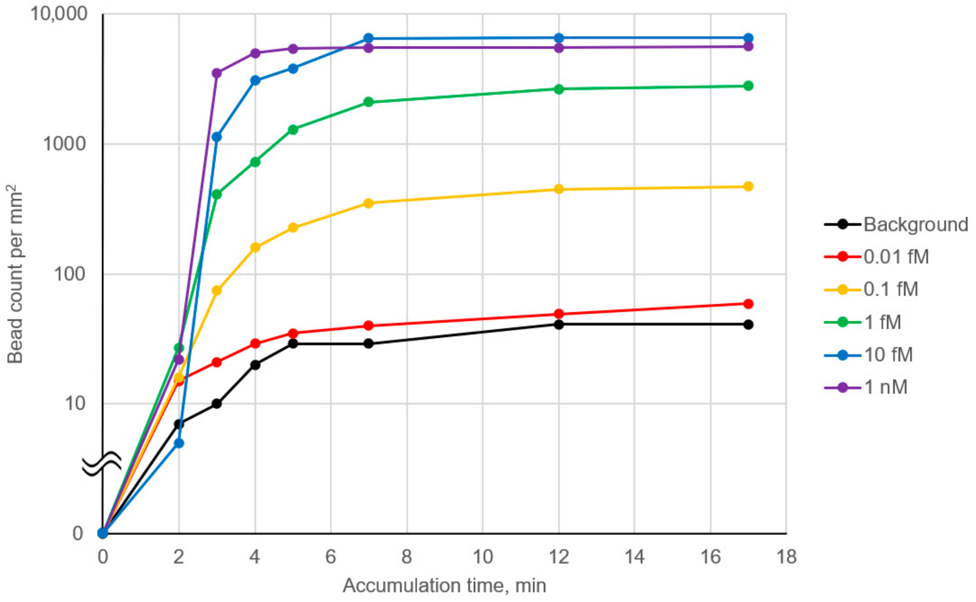

Figure S3. SEB signals and background accumulation kinetics for the indicated analyte concentrations. Error bars have been omitted for clarity of viewing. Typical signal variations can be assessed from the data in Fig. 3. The dead-volume passing time was 2 min.

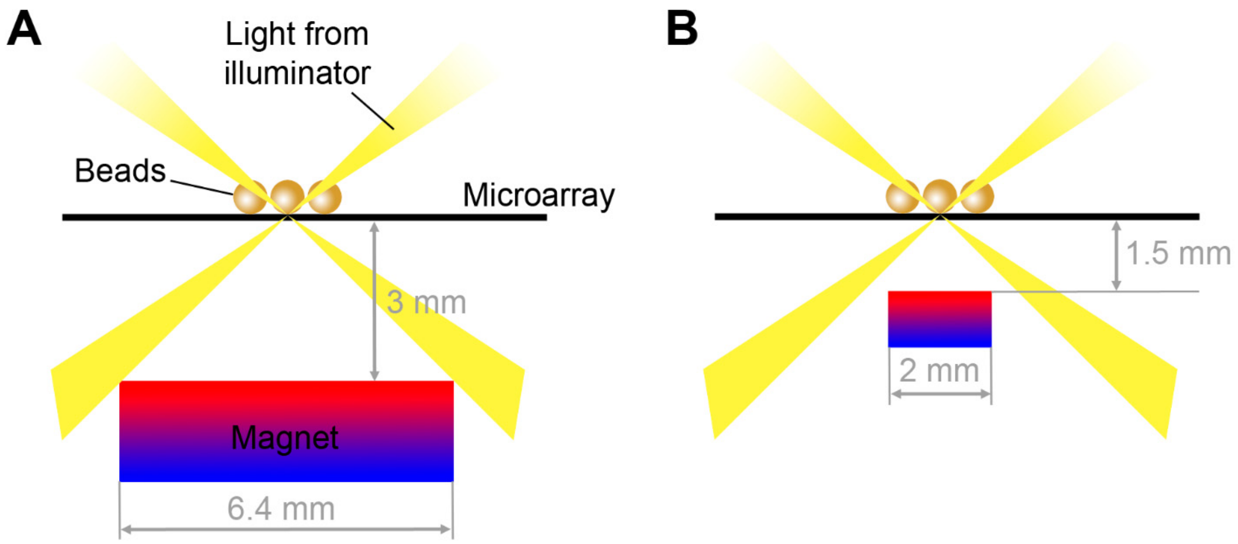

Figure S4. Designs of magnet placement in the microarray flow cell: (A) a previously described geometry [18] with a large magnet; (B) the geometry with a small magnet used in this work.

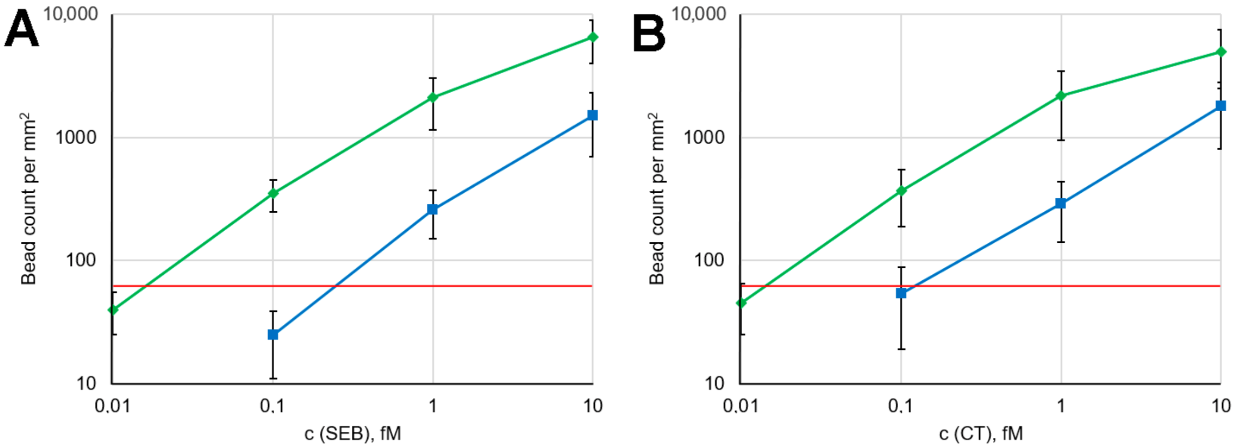

Figure S5. Calibration curves obtained with a large magnet (blue line): (A) SEB; (B) CT. For comparison, data obtained using a small magnet are shown (green line, same dataset as in Fig. 3). The red line marks the mean background + 2.5 STD, which is the same for both experimental setups within 10% accuracy. Error bars correspond to 2.5×STD.

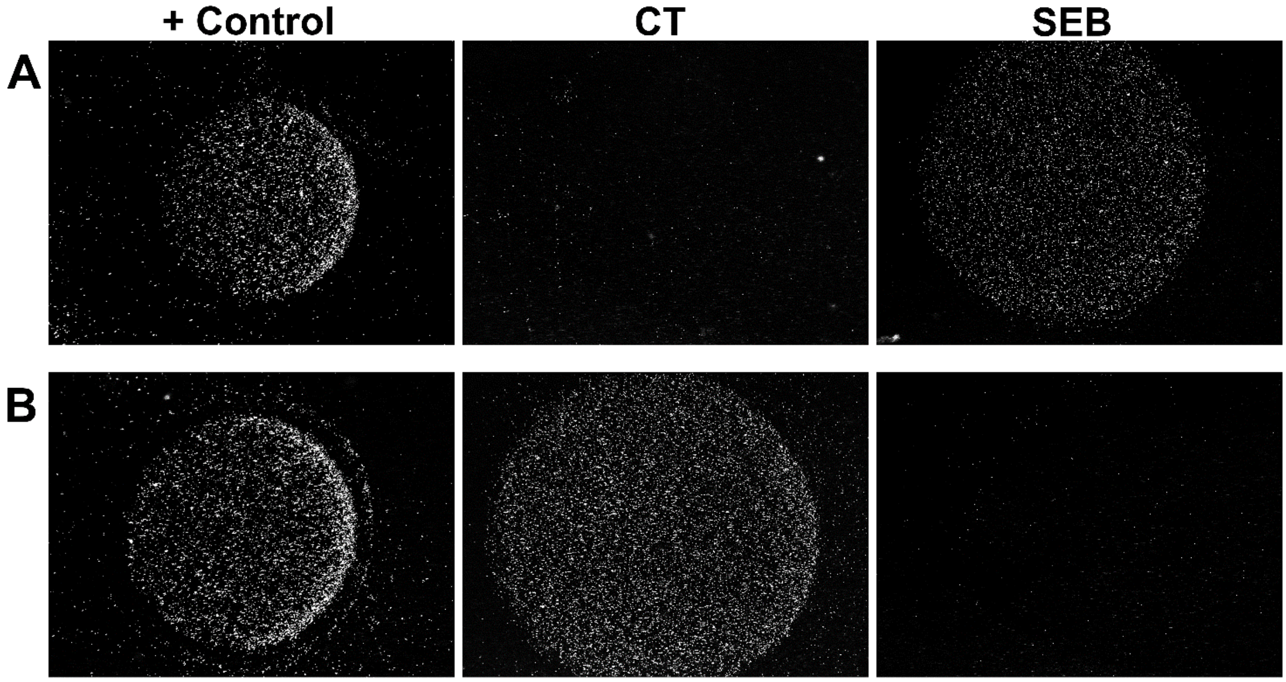

Figure S6. An illustration of the assay specificity. Assay results are shown for samples containing 1 nM SEB (A) or 1 nM CT (B). Each image corresponds to an area of 1.3 × 1 mm^2.
